# The influential role of personal advice networks on general practitioners’ performance: a social capital perspective

**DOI:** 10.1186/s12913-017-2467-x

**Published:** 2017-08-08

**Authors:** Stefano Calciolari, Laura G. González-Ortiz, Federico Lega

**Affiliations:** 1 Università della Svizzera Italiana, IdEP Via G. Buffi 13, CH-6904 Lugano, Switzerland; 2 Dept. Policy Analysis and Public Management, CERGAS, SDA Bocconi School of Management Bocconi University, Via Roentgen 1, 20136 Milan, Italy

**Keywords:** Social capital, Primary care, Goals, Italy

## Abstract

**Background:**

In several health systems of advanced countries, reforms have changed primary care in the last two decades. The literature has assessed the effects of a variety of interventions and individual factors on the behavior of general practitioners (GPs). However, there has been a lack of investigation concerning the influence of the resources embedded in the GPs’ personal advice networks (i.e., social capital) on GPs’ capacity to meet defined objectives.

The present study has two goals: (a) to assess the GPs’ personal advice networks according to the social capital framework and (b) to test the influence of such relationships on GPs’ capacity to accomplish organizational goals.

**Methods:**

The data collection relied on administrative data provided by an Italian local health authority (LHA) and a survey administered to the GPs of the selected LHA. The GPs’ personal advice networks were assessed through an ad-hoc instrument and interpreted as egocentric networks. Multivariate regression analyses assessed two different performance measures.

**Results:**

Social capital may influence the GPs’ capacity to meet targets, though the influence differs according to the objective considered. In particular, the higher the professional heterogeneity of a GP personal advice network, the lower her/his capacity is to meet targets of prescriptive appropriateness.

**Conclusions:**

Our findings might help to design more effective primary care reforms depending on the pursued goals. However, further research is needed.

## Background

Demographic and epidemiologic trends as well as health technology advances have been reshaping the configuration of health service delivery to better cope with emerging health needs. In the last two decades, reforms in several European health systems have changed primary care, for instance, modifying the organizational role of general practitioners (GPs) and the content of their activities [1, 2]. Primary care reforms have been aimed at fostering evidence-based medical practices as a strategic means to accomplish organizational and system goals.

The literature has investigated the impact of a variety of interventions and individual factors on GP behavior [3]. In particular, a traditional topic of investigation concerns the comparison between single handed GPs (or “solo” practices) and team practices, in terms of prescribing behavior and other outcome variables [2, 4]. A recent research topic regards the implications of relationships created through institutional collaboration initiatives on GP performances [1, 5]. The basic idea behind this line of enquiry is that the relationships nurtured by GPs within collaborative arrangements forge networks influencing GP professional behavior; in particular, such networks are expected to enhance the GPs’ capacity to contain pharmaceutical costs or to meet other objectives.

However, the GPs’ personal network of relationships (e.g., entrusted colleagues available to be contacted for advice) might also significantly influence GP decision-making [6]. In fact, the rising pressures associated with new accountability systems call for timely reactions to uncertainty. In such circumstances, informal advice relationships can be valuable to cope with the growing knowledge complexity of medical science and new health needs that call for interpretive action and interaction [7].

Consequently, building on a social capital perspective, our goal is twofold: (a) to assess the personal advice relationships developed by GPs with other health professionals in their Local Health Authority (LHA); and (b) to test the influence of these relationships on GPs’ capacity to meet two different organizational objectives established by their LHA and concerning their prescribing behavior: containing pharmaceutical costs and meeting prescriptive standards.

### Theoretical model

Clinical knowledge is surrounded by a certain level of uncertainty and ambiguity [7]. For instance, there is disagreement among professionals about what constitutes scientific evidence [8]. In addition, even shared scientific evidence often lacks applicability to specific contexts of everyday practice [9, 10]. Consequently, in situations where the human capital (i.e., individual stock of competences and knowledge) is not sufficient to reduce the aforementioned uncertainty, professionals evaluate and interpret clinical knowledge by interacting with colleagues. In other words, they rely on social capital to search for clues to reduce the risks – actual or perceived – associated with uncertainty. In fact, the literature concerning influences on GP prescribing behavior deals extensively with social factors, though mainly in descriptive terms.

Social capital can be defined as the actual and potential resources that pertain to a subject (actor or node) through its network of relationships [11], although the actor does not have exclusive ownership of such resources [12]. In fact, social capital theory posits that a node can access the resources embedded in its relationships [13, 14].

In our study, we focus on relationships that GPs establish when they informally look for advice from other health professionals. We argue that information and influence, embedded in these relationships, trigger GPs’ prescribing behavior and, consequently, impact their capacity to meet goals related to this behavior. In particular, connections facilitate a focal actor’s (ego) access to broader sources of information and improve information’s quality, relevance, and timeliness [12, 15, 16]. Therefore, advise-seeking relationships might help a GP to acquire pertinent, evidence-based, update information in the first instance.

However, relationships (or tie) can have diverse qualifications thus differentiating the network they compose. We based the notion of network heterogeneity on the concept of boundary. A tie can span different types of boundaries (e.g., physical, organizational, professional). Echoing the distinction between “internal” and “external” ties [17], we build on the idea that, respectively, intra- and inter-professional links provide the focal actor with distinct resources. In particular, boundary-spanning ties (or bridging ties), linking actors who belong to different professions, are important conduits of information and capabilities [18, 19].

In this respect, Nair et al. [20] found that GPs were likely to be influenced by the prescribing behavior of specialist physicians (or “opinion leaders”) in their network when an exogenous phenomenon occurs (e.g., a new guideline or product), but the influence does not work in the opposite direction. They called this phenomenon the “asymmetric peer effect*”*, which identifies the prominent influence of specialists on GPs’ prescribing behavior. Therefore, heterogeneous advice networks are conduits of influence that might weaken the focal actor’s distinctive professional goals and norms through the mechanisms of social contagion or prominence [21]. This phenomenon does not assume disagreement between GPs and specialists, but suggests that, when GPs are uncertain on treatment and ask for advice, specialists tend to conform GPs’ prescriptive behavior to their own and, thus, away from guidelines that apply only for GPs.

In conclusion, we argue that, everything else equal, the composition and heterogeneity of a GP’s personal advice network influence her/his capacity to meet organizational objectives, because her/his personal advice network is a proxy of the individual-level social capital that a GP can activate to reduce the uncertainty surrounding her/his decision-making.

## Methods

### Aim of the study

The main goal of our study consists of assessing the influence of a GP’s social capital on her/his capacity to accomplish two organizational objectives related to his/her prescribing behavior: containing health expenditures and fostering prescriptive standards.

Building on the notions of social contagion and asymmetric peer effect, we expect that GPs seeking advice from different health professionals are less likely to meet organizational goals linked with their gatekeeping function compared with GPs relying on mono-professional links.

*HP1: The heterogeneity of a GP’s personal advice network negatively influences her/his ability to contain costs and meet prescriptive standards.*

Following the general notion that better access to information improve an actor’s capacity to meet goals (because, for instance, reduces uncertainty), we expect that GPs with larger advice networks are more likely to meet organizational goals.

*HP2: The size of a GP’s personal advice network positively influences her/his ability to contain costs and meet prescriptive standards.*

### Empirical setting of the study

In the Italian National Health System, local health authorities (LHAs) are responsible for the health of the population residing in their area and for managing GPs, who are independent contractors and are paid mainly with a capitation system. They act as gatekeepers to drugs and higher levels of care, and citizens cannot contact specialists without consulting a GP, unless they are willing to pay out-of-pocket. Each patient can choose among the GPs working in the city of residence.

Based on the idea that organizational and professional development requires cooperation and the sharing of resources in primary care, several LHAs have implemented collaborative arrangements in which GPs exchange knowledge and share practice space and other resources [2]. In addition, LHAs have introduced information systems and incentive mechanisms to make GPs more accountable for their prescribing and referral behaviors [1].

The study focuses on the LHA of Forlì, in the Emilia-Romagna Region of Italy, which serves a total population of 187,698. The LHA regularly collects data on GP membership and prescribing behavior. We use data provided by the LHA from its administrative records, and data collected in the field with an instrument administered to the GPs.

Since 2012, 139 GPs were working for the LHA and all were engaged in collaborative arrangements called *nuclei di cure primarie* (primary care groups – PCGs). A PCG identifies a group of GPs who are subject to performance accountability systems focused on two types of objectives: (a) cost containment of pharmaceuticals (henceforth referred to as *economic objective*); and (b) promotion of compliance with selected prescriptive standards (henceforth referred to as *prescriptive appropriateness objective*). Each type of objective is associated with two financial incentives, one concerning the accomplishment of individual targets and one concerning targets at the PCG-level. This paper focuses on the individual targets.

### Variables

#### Dependent variables

The first outcome variable concerns the economic objective, which consists of not exceeding the average annual pharmaceutical expenditure per capita in the Region. This objective was established by the LHA through a contractual agreement with the GPs, which basically recognized that no relevant epidemiologic reason could justify a significant deviation above the regional average expenditure. In our study, this variable was expressed by the difference between the LHA’s assigned target and the individual GP’s per capita pharmaceutical expenditure (i.e., the higher the difference the better the performance).

The second dependent variable regards the objective of prescriptive appropriateness, which consists of six targets, regarding different pharmaceutical categories: proportion of prescribed generics for statins, drugs for osteoporosis (ATC^1^ code M05), and angiotensin II-receptors blockers (ARB) not below the regional average; daily doses per 1000 inhabitants for selective serotonin reuptake inhibitors (SSRI) not above the regional average; proportion of an angiotensin-converting enzyme inhibitors (ACEI) over the sum of ACEI and ARB prescribed above the regional average; and proportion of BPCO^2^/Asthma daily doses prescribed not associated above the regional average. Even this objective was established via contractual agreement between the LHA and the GPs, based on the consideration that the epidemiologic characteristics of the LHA’s territory could be assimilated to the regional average. The rationale for increasing the proportion of prescribed generics consist of fostering the use of drugs widely tested, not infrequently recommended as the first choice by international organizations, and scoring high in the cost-effectiveness ranking when compared with alterative drugs. The rationale of the other targets consists of fostering the standardization of the GPs’ prescribing behavior across populations epidemiologically similar. In our study, this second objective was expressed as the sum of the accomplished targets, thus defining an ordinal variable with values ranging from 0 to 6. Implicitly, we do not assume any priority between targets because no target resulted more important than another in the adopted incentive scheme. However, meeting more targets result in higher incentives for a GP.

#### Independent variables

The literature only recently started analyzing the influence of networks in healthcare, though it has investigated the impact of a variety of interventions and individual factors on GP behavior. Therefore, we accounted for the main influential variables in this respect.

First of all, the logistic arrangements aimed to push GPs toward team practices, and the team size is likely to influence a GP’s behavior because team-based approaches to patient care foster interactions between professionals [2, 22] and are important components of quality improvement [23]. Therefore, our analysis took into consideration the different extent to which GPs share their practice space and the size of the teams defined by the LHA.

In this respect, ten out of eleven PCGs have a reference location of practice where a patient can receive ambulatory services that are urgent but not serious enough to justify access to emergency service. In a reference location, one or more GPs have their practice and arrange their office hours with the members of their PCG to ensure that patients enjoy extended daily access (usually 12 h, except for the weekend) to primary care with one of the PCG members. Consequently, any GP can work according to one of the following three modalities: (a) exclusively in a solo practice; (b) in a solo practice but also working part-time in the PCG reference location – hereafter referred to as “partially shared” practice; (c) only in the PCG reference location of practice – hereafter referred to as “fully shared” practice. Independent from the working modality, any GP is member of a PCG and accountable to the aforementioned performance system. Except for the logistic arrangements, the organizational arrangements implemented in the PCGs are the same.

Size and demographic characteristics of the GP patient list are further aspects deserving attention. In fact, previous studies showed that the number of patient assisted is a relevant control factor when studying GPs’ prescriptive behavior [24, 25], and it is well known that elderly patients use a higher amount of health resources compared with younger age groups [26].

Another influential factor is the population density because the level of urbanization was found to be associated with significant differences in GP prescribing behaviors [27, 28].

### Data and materials

We relied on archival sources of the LHA to collect data on: (a) GPs’ capacity to meet the two aforementioned objectives (i.e., dependent variables); (b) the individual characteristics considered (e.g., gender, number of patients assisted, proportion of elderly patients assisted); (c) the characteristics of each PCG (e.g., team size, population density); (d) the logistic arrangement characterizing each GP practice, and the expected expenditure adjustment index.^3^

We used an ad-hoc instrument to collect data on the GPs’ direct advice relationships with other health professionals. The questionnaire asks respondents (ego) to disclose their names, while – to protect confidentiality – they were allowed to use initials or special codes to name their different personal contacts (alters), who were explicitly classified by profession (e.g., GP, nurse, psychologist). Specifically, we asked GPs (considering the last 12-month period): “If you need to discuss what the best treatment is for a patient, who do you call?” Respondents could indicate up to six unique key contacts (direct ties) forming their personal advice networks (or ego-networks).

Paper-and-pencil questionnaires were administered during LHA training initiatives targeting all its GPs in the period March–April 2013. We collected 80 usable questionnaires (58% response rate) and performed a test for nonresponse bias to check whether our results were affected by unknown factors that systematically distinguished respondents from non-respondents [29]. We compared the proportions or the means between respondents and non-respondents for seven variables: GP gender, age, number of patients assisted, proportion of elderly (i.e., aged over-75) patients assisted, expected expenditure adjustment index, population density in the PCG geographic areas, PCG team size, economic performance, and prescriptive appropriateness performance. We found no sign of nonresponse bias at the 95% confidence level.

We considered three measures to assess the GP personal advice network: the number of ties (or degree centrality), Index of Qualitative Variation (IQV), and the E-I index. The measures assess, respectively, the composition, heterogeneity and homophily of a personal network [30]. The formulas of the last two measures are reported in (1) and (2), respectively.

1 $$ {IQV}_{\mathrm{i}}\kern0.5em =\kern0.5em {\mathrm{k}}_{\mathrm{i}}\kern0.15em \left({100}^2,-,\sum_1^{ki},{Pct}^2\right)/{100}^2\kern0.15em \left({\mathrm{k}}_{\mathrm{i}}-1\right) $$

Where *i* is the ego, *k*_*i*_ is the number of professional categories in the network of *i* and Pct is the percentage of the *k* professional category in distribution of alters for *i*. In our case, the IQV measures the professional alters’ diversity in each egocentric network, with 0 indicating uniformity and 1 the maximum observed diversity.

2 $$ E\hbox{-} I\kern0.1em {index}_{\mathrm{i}}\kern0.5em =\kern0.5em \left({\mathrm{E}}_{\mathrm{i}}-{\mathrm{I}}_{\mathrm{i}}\right)/\left({\mathrm{E}}_{\mathrm{i}}+{\mathrm{I}}_{\mathrm{i}}\right) $$

Where *i* is the ego, *E*_*i*_ is the number of *i*’s ties with alters belonging to professional categories other than GP (i.e., bridging ties), and *I*_*i*_ is the number of *i*’s links with alters who are GPs (i.e., bonding ties). The E-I index measures the extent to which an ego’s ties form a bridge across some social divide [31]. In our case, we consider the divide originated from belonging to different health professions. The measure ranges from −1 (in our case, when *i* all have ties with GPs) to 1 (when all ties to/from *i* are bridging ties).

### Analysis

We analyzed our data using two multivariate regression models, coherently with each type of dependent variable: a log-linear OLS regression model for the economic performance (the logarithmic transformation aims to better approximate normality in the distribution of the dependent variable) and an ordinal logistic regression model for the prescriptive appropriateness.

Analyses were performed using E-Net (version 0.41) and Stata (version 12) software packages.

## Results

Table 1 shows the descriptive statistics of the variables used in the analysis. The average GP is male (while 46% of GPs are female), approximately 56 years old, and assists approximately 1278 patients (14% of whom are over 75).

**Table 1:** Descriptive statistics

Variable	Obs	Mean/Prop	Std. Dev.	Min	Max
Economic performance	79	−10.91	37.31	−87.65	115.44
Prescriptive appropriateness	80	1.73	1.24	0	5
Age	77	55.66	6.04	33	67
Gender (M = 1)	80	0.64	-	0	1
N. patients (log-transformed)	80	6.95	1.06	0.69	7.44
Proportion elderly patients (75+)	80	0.14	0.04	0.00	0.24
Expected expenditure adjustment index	79	0.91	0.17	0.70	1.75
Population density (PCG level)	80	353.81	199.02	32.08	521.33
PCG teamsize	80	13.95	4.32	5	20
N. Ties	80	3.20	1.35	1	6
IQV (profession)	80	0.85	0.34	0.00	1.00
E-I index (profession)	80	0.26	0.52	−1.00	1.00
Solo practice	80	0.45	-	0	1
Partially shared practice	80	0.18	-	0	1
Fully shared practice	80	0.38	-	0	1

*PCG* Primary care group, *IQV* Index of Qualitative Variation (based on the variable “profession”), *E-I* External – Internal

The majority of the respondents (approximately 55%) work in a team practice (either having a single, common practice or working part-time in a PCG reference location), while 45% of them work exclusively in a solo practice.

There is a relevant variability of the population density (from approximately 32 to 521 inhabitants per squared kilometer) in the geographic areas served by the eleven PCGs.

On average, GPs are not able to meet the economic objective (mean difference = −10.91€ per-capita) when their expenditure is not adjusted to take into account the age and gender of the patients enrolled with each GP, and they meet 1.73 (out of six) targets of prescriptive appropriateness.

Table 2 synthetically describes the main characteristics of the respondents’ personal advice networks. On average, a GP consults 3.2 informal contacts when she/he needs to discuss an important professional issue or what the best cure is for a patient, and only one contact is a GP.

**Table 2:** Descriptive statistics of the GPs’ personal advice networks (ego-alter ties)

Variable (alter)	Obs	Mean	Std. Dev.	Min	Max
GP	80	1.06	0.43	0	2
Psychologist	80	0.75	0.44	0	1
Nurse	80	0.81	0.53	0	2
Cardiologist	80	0.13	0.33	0	1
Hospital Specialist (excluding Cardiologist)	80	0.45	0.57	0	2
*non GP*	*80*	*2.14*	*1.25*	*0*	*5*
*any professional*	*80*	*3.20*	*1.35*	*1*	*6*

*GP* General practitioner

The observed variability of the networks is relevant in terms of composition (12.5% of the respondents reported one tie, while approximately 64% reported up to three ties and only 6.3% reported the maximum number of ties – i.e., six – observed), professional heterogeneity (only 13.5% of the observed network is mono-professional and approximately 68% displays the highest professional heterogeneity observed – i.e., IQV = 1), and professional homophily (approximately 11.3% of respondents reported links only with other GPs, approximately 6.3% did not report a single link with another GP, and 7.5% reported an equal number of GP and non-GP informal contacts).

Our main goal was to test the relationship between the characteristics of a GP’s personal advice network and her/his capacity to meet the two organizational goals mentioned above.

As far as the economic objective is concerned, Table 3 shows the results of the first regression model. Controlling for the expected expenditure adjustment index, that reflects the characteristics in terms of age and gender of the assisted patients, the size of the assisted population is negatively associated with the GP’s economic performance. In particular, a 10% increase in the number of assisted patients is associated with a decrease in the performance equal to 2.2€ per-capita (i.e., −23.003*ln(1.1)), which is over 20% of the average performance (i.e., −10.91€ per-capita).

**Table 3:** Explanatory model for the economic objective

Variable	Coef.	Std. Err.	[95% Conf. Interval]	
Age	0.237	0.579	−0.920	1.394
Gender (M = 1)	- 6.792	6.829	−20.434	6.851
N. patients (log-transformed)	- 23.003^b^	3.899	−30.791	−15.215
Exp. expenditure adj. Index	130.223^b^	19.438	91.391	169.054
Population density (PCG level)	0.001	0.037	−0.073	0.076
PCG teamsize	1.451	1.688	−1.922	4.824
*Personal advice network*				
Size (number of ties)	- 1.068	2.813	−6.688	4.552
Heterogeneity (IQV, profession)	5.466	13.386	−21.277	32.208
Homophily (E-I index, profession)	3.282	8.358	−19.980	13.416
*Logistic arrangement*				
Solo practice *– omitted*	-	-		
Partially shared practice	18.232^b^	8.790	0.673	35.791
Fully shared practice	10.886^a^	6.587	−2.273	24.045
*N* = 76 F(11) = 9.11 Prob > chi2 = 0.000 R-squared = 0.610 Adj. R-squared 0.543				

^a^90% confidence level; ^b^95% confidence level

*Exp. expenditure adj. Index* Expected expenditure adjustment index, *PCG* Primary care group, *IQV* Index of Qualitative Variation (based on the variable “profession”), *E-I* External – Internal

The characteristics of the GP personal advice network did not seem to influence her/his capacity to meet the economic objective, while there is evidence that the logistic arrangements do influence such capacity. Compared with solo practice, team practice is associated with significantly better economic performance. In particular, GPs who partially or fully share their practice enjoy a higher per-capita performance, respectively, 18.2€ or 10.9€.

As far as the prescriptive appropriateness objective is regarded, Table 4 shows the results of the ordinal logistic regression model. Interestingly, the population density of the area served by the PCG is negatively associated with the GP’s capacity to comply with prescriptive standards; for a one-unit increase in population density, the odds of accomplishing at least one target versus no target are 1% lower, given that the other variables in the model are held constant.

**Table 4:** Explanatory model for the prescriptive appropriateness objective

Variable	Odds ratio	Std. Err.	[95% Conf. Interval]	
Age	1.022	0.043	0.939	1.113
Gender (M = 1)	1.432	0.498	0.540	3.799
N. patients	1.000	0.001	0.999	1.001
Proportion elderly patients (75+)	0.041	5.174	0.000	1029.555
Population density (PCG level)	0.993^b^	0.003	0.988	0.999
PCG teamsize	1.256^a^	0.129	0.975	1.617
*Personal advice network*				
Size (number of ties)	0.936	0.208	0.622	1.409
Heterogeneity (IQV, profession)	0.111^b^	0.967	0.017	0.737
Homophily (E-I index, profession)	1.638	- 0.575	0.531	5.057
*Logistic arrangement*				
Solo practice *– omitted*	-	-		
Partially shared practice	1.871	0.640	0.534	6.555
Fully shared practice	0.396^a^	0.498	0.149	1.049
*N* = 77 LR chi2(11) = 21.24 Prob > chi2 = 0.031 Log likelihood = −111.586 Pseudo R2 = 0.087				

^a^90% confidence level; ^b^95% confidence level

*PCG* Primary care group, *IQV* Index of Qualitative Variation (based on the variable “profession”), *E-I* External – Internal

The GP’s personal advice network influences her/his performance in terms of meeting prescriptive standards. However, the size of the network is not influential. While a GP with a heterogeneous personal network is likely to accomplish fewer targets compared to a GP with a homogeneous one. In particular, the odds of accomplishing at least one target versus no target are 0.11 times those of a GP with the most heterogeneous informal advice network (i.e., IQV = 1) compared to a GP with the most homogeneous network (i.e., IQV = 0), everything else being equal (CI95%). In terms of changes in probability, the average GP with a given personal advice network characterized by given degree of homogeneity (e.g., IQV = *q*, with 0 ≤ *q* < 1) is about 30 and 25 percentage points less likely to meet, respectively, none or only one target compared to the average GP with a more heterogeneous network (e.g., IQV = *q* + δ, with δ close to zero); while the former type of GP is consistently more likely to meet two or more targets (with marginal effects at the means ranging from about 2 to 25 percentage points) compared with the latter one.

Finally, there is mixed evidence about the influence of logistic arrangements on GP prescriptive appropriateness. On average, the odds of accomplishing at least one target versus no target are 0.40 times those of a GP who fully shares his/her practice compared to a solo practice (CI90%), everything else being equal, while there is no evidence of a significant difference in the odds associated with GPs who partially share their practice compared to GPs working exclusively in solo practices.

In conclusion, the results partially support our first hypothesis, while they do not support our second hypothesis.

## Discussion

The variability observed in the personal advice networks is not related to individual GP factors or the logistic arrangements implemented to foster team practice. In fact, we tested the relationship between the network measures and the other independent variables in our two explanatory models by means of multivariate regression; the results show no sign of a statistically significant association between the independent variables and the network measures included in the two models. Therefore, it is likely that the characteristics of the personal networks depend on the different strategies that each GP deliberately adopts to work out appropriate decisions in uncertain situations.

The analysis of two different targets highlighted that a GP’s personal advice network plays different roles according to the objective considered. In particular, when an economic objective is considered, holding everything else constant, the logistic arrangements fostered by a team practice have a positive influence on a GP’s capacity to contain expenditures, while the GP’s personal advice network does not influence her/his economic performance. On the contrary, when targets concerning prescriptive appropriateness are considered, the GP’s personal advice network is influential to her/his capacity to meet them.

The information collected with the qualitative interviews conducted during the design of the instrument along with our preliminary results support a possible explanation. The performance concerning the first type of objective is influenced by a variety of management factors (such as the GP’s ability to persuade patients to purchase the prescribed drugs from the closest hospital pharmacy or being frequently updated about drug patent developments) that are also likely to be influenced by the GPs’ relationships with their peers rather than other health professionals.

In this respect, the logistic arrangements may capture a relevant part of the relationship among peers, as well as the coordination efforts among the GPs (e.g., adopting uniform communication strategies with patients, sharing information about patients’ medical records) and between the GPs and the LHA (that also manages the public local hospitals). Inversely, the target regarding the second type of objective might be most influenced by the GP’s social interactions with specialists or “opinion leaders” because, especially when changes occur in the therapeutic environment (e.g., pharmaceutical innovations or introduction of new guidelines), GPs solicit the opinions of specialists when making prescribing decisions [20].

As far as the direction of such a significant influence is concerned, we already mentioned that the mechanisms of social influence operating among GPs might differ from those operating between GPs and other health professionals. In particular, the interaction with specialists may emphasize the benefits of innovation at the expense of the GP’s goals associated with their gatekeeping function and norms (e.g., implement prescriptive standards); Nair and colleagues’ [20] concept of “asymmetric peer effect” intuitively suggests the dynamics associated with this type of social interaction. According to this line of reasoning, the information and advice flowing from a heterogeneous personal network are likely to conflict with the GP’s goals and negatively influence her/his capacity to accomplish the prescriptive targets.

### Practical implication

The results suggest interesting policy/management implications. Because GPs need to interact with colleagues to reduce uncertainty, fostering training and/or communication initiatives to share primary care goals among other health professionals is paramount. In particular, it is important to design strategies aimed at aligning the objective of prescriptive appropriateness among the different health professionals. Like the pharmaceutical industry invested in targeting marketing activities at opinion leaders among physicians [20], so health authorities could invest in initiatives aimed to raise the awareness regarding appropriate prescribing behaviors (e.g., shared guidelines and care protocols) and eventually incentivize multi-professional cooperation toward enhancing prescriptive appropriateness.

The positive influence of team practice approaches on a GP’s capacity to contain pharmaceutical expenditures suggests that logistic arrangements are effective at supporting cost control strategies and facilitating the accomplishment of individual targets linked to this type of organizational goal. However, team practice approaches are not influential in the accomplishment of other types of goals. In other words, there is not a one-size-fits-all management solution in primary care reforms.

Finally, the negative influence of population density on GPs’ prescriptive appropriateness may indicate that GPs are better able to implement prescribing changes in rural/countryside areas, compared with urban centers, perhaps due to the higher authority they enjoy with their patients. In fact, with highly mobile populations and a plentiful supply of doctors, regulations for access and use of services are more difficult to maintain in urban centers compared with the countryside [32]. This is an important aspect to consider when designing strategies aimed at reducing the inappropriate use of drugs.

## Conclusions

Two limitations of the study are the sample size and the absence of information about chronic conditions. As far as the former is concerned, despite the good response rate and the absence of relevant discontinuities in the primary care of the investigated LHA, caution should be used when generalizing our results. In addition, we could not collect data about the prevalence of chronic conditions at GP level. However, because aging is associated with chronic illnesses, we consider the variables concerning assisted patients aging (i.e., proportion of elderly and expected expenditure adjustment index) to be rough proxies of this factor in our models.

We found that GPs’ personal advice networks are significantly different. The variability of the social capital inherent to GPs’ personal advice networks influences her/his capacity to accomplish specific types of organizational goals. In particular, a GP’s propensity to ask for advice from different professionals works as a detriment to her/his capacity to comply with prescriptive standards, controlling for the GP’s individual factors and the team-based approaches to primary care practice. However, the characteristics of a GP personal advice network did not influence her/his capacity to contain pharmaceutical expenditures. Our study is based on cross-sectional data from one LHA of the Italian NHS. Therefore, results should be generalized with caution, though their policy and managerial implications might help health authorities design more effective primary care reforms. Further research could use longitudinal data to test the causality of the discussed relationships. However, an eventual extension of our study must consider the LHA of Forlì merged with another LHA in 2013 and is currently an autonomous district of the new LHAs of Romagna. Alternatively, qualitative in depth analyses could better explain the micro-dynamics behind our findings.

## Abbreviations

GP: General Practitioner
IQV: Index of Qualitative Variation
LHA: Local Health Authority
OLS: Ordinary Least Squares
PCG: Primary Care Group

## Footnotes

1 Anatomical Therapeutic Chemical Classification System. The code “M05” identifies drugs for treatment of bone diseases.

2 Broncho-Pneumopathie Chronique Obstructive.

3 This index is computed by the LHA taking into account the age and gender of the patients enrolled with each GP. When the index is higher/lower than 1, it means that the average patient of a particular GP is more/less expensive than the average patience of the population.
